# Correction of motion‐induced susceptibility artifacts and B_0_ drift during proton resonance frequency shift‐based MR thermometry in the pelvis with background field removal methods

**DOI:** 10.1002/mrm.28302

**Published:** 2020-05-05

**Authors:** Mingming Wu, Hendrik T. Mulder, Paul Baron, Eduardo Coello, Marion I. Menzel, Gerard C. van Rhoon, Axel Haase

**Affiliations:** ^1^ Munich School of Bioengineering, TUM Department of Physics Technical University of Munich Garching Germany; ^2^ Erasmus MC Cancer Institute Rotterdam the Netherlands; ^3^ GE Healthcare Munich Germany

**Keywords:** B_0_ drift, background field removal, hyperthermia, motion, MR thermometry, susceptibility

## Abstract

**Purpose:**

The linear change of the water proton resonance frequency shift (PRFS) with temperature is used to monitor temperature change based on the temporal difference of image phase. Here, the effect of motion‐induced susceptibility artifacts on the phase difference was studied in the context of mild radio frequency hyperthermia in the pelvis.

**Methods:**

First, the respiratory‐induced field variations were disentangled from digestive gas motion in the pelvis. The projection onto dipole fields (PDF) as well as the Laplacian boundary value (LBV) algorithm were applied on the phase difference data to eliminate motion‐induced susceptibility artifacts. Both background field removal (BFR) algorithms were studied using simulations of susceptibility artifacts, a phantom heating experiment, and volunteer and patient heating data.

**Results:**

Respiratory‐induced field variations were negligible in the presence of the filled water bolus. Even though LBV and PDF showed comparable results for most data, LBV seemed more robust in our data sets. Some data sets suggested that PDF tends to overestimate the background field, thus removing phase attributed to temperature. The BFR methods even corrected for susceptibility variations induced by a subvoxel displacement of the phantom. The method yielded successful artifact correction in 2 out of 4 patient treatment data sets during the entire treatment duration of mild RF heating of cervical cancer. The heating pattern corresponded well with temperature probe data.

**Conclusion:**

The application of background field removal methods in PRFS‐based MR thermometry has great potential in various heating applications and body regions to reduce motion‐induced susceptibility artifacts that originate outside the region of interest, while conserving temperature‐induced PRFS. In addition, BFR automatically removes up to a first‐order spatial B_0_ drift.

## 1 INTRODUCTION

Mild hyperthermia (HT) treatment (40‐44°C) of different types of cancer is applied to sensitize tumor cells to radiotherapy and chemotherapy.^1^ For mild HT of the pelvis, radio frequency (RF) hyperthermia (RF‐HT) provides both deep penetration depth and a large enough tissue coverage during heating. Targeted anatomies include cervical, rectal, bladder, and prostate cancer. Even though invasive temperature probes are still commonly used to ensure sufficient thermal exposure in the target tissue, efforts are made to monitor temperature with interventional MRI with the advantage of a larger volume coverage and a higher spatial resolution. However, because common MR thermometry methods rely on a reference scan, the methods are very sensitive to any interscan motion.

The most widespread method for MR‐based temperature monitoring during mild RF‐HT is based on the linear proton resonance frequency shift (PRFS) of water molecules with temperature.^2^, ^3^, ^4^ By calculating the phase difference between a gradient echo image at a certain temperature and a reference temperature, the temperature change is deduced. Double Echo Gradient Echo (DEGRE) is used for monitoring mild HT of tumors in the pelvis^2^, ^5^ and sarcomas.^6^ DEGRE can correct for conductivity change‐induced phase bias^7^ but remains prone to other phase change confounders,^8^ including motion‐induced susceptibility artifacts. Motion within the pelvis that originates from muscle contraction and relaxation, or involuntary bulk motion, will lead to actual tissue displacement. If the region of interest (ROI) is displaced, the reference method cannot be applied any longer. Displacement of tissue outside the ROI originating from breathing or digestive motion also alters the underlying macroscopic magnetic field with time, which would be misinterpreted as temperature change. The alteration of the magnetic susceptibility distribution induces a field change equivalent to the susceptibility difference convoluted with a dipole. Because the dipole propagates into neighboring tissue, large errors in temperature maps are induced.^5^, ^9^ We first investigated the impact of breathing on the magnetic field change within the pelvic region inside and outside the RF applicator in the presented work.

The confounding effect of temperature‐induced susceptibility change on PRFS‐based MR thermometry has been investigated before. One study revealed that the change of the surrounding air temperature changes the air susceptibility. A temperature rise of air of 46°C led to a temperature error of up to 1.9°C in the phantom.^10^ Furthermore, the susceptibility change of fat with temperature has been addressed as a potential confounder for MR thermometry during thermal therapies in neighboring muscle tissue.^11^ In the context of MR thermometry‐guided high‐intensity focused ultrasound (MRgHIFU), where the temperature rise induced a susceptibility change of fat, the bias was corrected by forward‐calculating the associated field change in the Fourier space and successively subtracting it from the measured field.^12^ In this case, the temperature rise of fat was first estimated with T1 maps. However, in this forward calculation approach, both the exact susceptibility change value as well as the geometry of the susceptibility change origin need to be known.

Digestive motion, where paramagnetic gases change position with diamagnetic water‐based tissues, implies a manifold higher confounding effect than the temperature change‐induced susceptibility artifacts. One work has addressed the digestive gas artifacts by applying a spatiotemporal filter^9^ on the phase images as a post‐processing step. However, it was falsely assumed that the artifact is spatially contained.

Removing the digestive susceptibility change‐induced field changes in the pelvis corresponds to the problem of removing the background field before calculating the magnetic susceptibility values from the local field during quantitative susceptibility mapping.^13^ In the context of MR thermometry, projection onto dipole fields (PDF)^14^ was already presented as a method to improve temperature accuracy in a phantom study.^15^ It was also applied to correct for susceptibility artifacts in human PRFS‐MR thermometry abdominal data at constant temperature,^16^ as well as during liver ablation of a pig.^17^ However, only a 2D slice was acquired, because the field changes and tissue displacement with breathing motion in the abdomen required a high temporal resolution. Among other background field removal (BFR) methods, solving the Laplacian boundary value (LBV)^18^ has shown to perform best in the kernel‐convolution‐based BFR methods,^19^ showing minimal artifacts in the internal field and no limitations compared to other BFR methods such as V‐SHARP,^20^ RESHARP,^21^ or HARPERELLA.^22^ Furthermore, LBV and PDF were recommended for data with large susceptibility variations.^23^

Another confounder of PRFS‐based MR thermometry is given by the B_0_ field drift with time caused by hardware heating and cooling. This is of interest for mild heating because the expected field drift is in the order of the resonance frequency shift with temperature during the treatment period of 90 minutes.^24^ One common approach for extracting and correcting for B_0_ field drift uses references outside the heated area and extrapolates a first‐order spatial field drift over the acquired image. External reference tubes were filled with oil or silicon.^25^, ^26^ Problems associated with external reference tubes include an increased imaging volume, low signal‐to‐noise‐ratio (SNR) of the reference signal, and thus low robustness of the correction method. In the context of deep mild RF‐HT, the reference tubes are placed in the periphery of the field of view, where it suffers from increased field inhomogeneity and thus a shorter $T_{2}^{*}$ and lower SNR. Alternatively, the patient’s adipose tissue signal was used as a reference.^26^ As discussed before,^11^, ^12^ this will only work if the fat tissue is not heated itself. A referenceless PRFS thermometry method was proposed to overcome field disturbances not originating from temperature by fitting a smooth plane onto the phase and excluding the area of heating.^27^, ^28^, ^29^ However, this method assumes a highly locally confined heating area, which does not apply for regional heating of mild RF‐HT.

The correction of the field drift for higher temperature accuracy gained attention for mild HT using MRgHIFU as well. A recent publication corrected the B_0_ drift by additionally acquiring nonselective free induction decay signals from multiple coils.^30^ Using field probes for drift correction was also proposed.^31^ Our suggestion to use BFR techniques will overcome these problems without the need of extra data acquisition or additional hardware.

In this work, we first quantify the expected scale and the spatial extension of motion‐induced temperature errors in the presence of digestive gas motion inside a MR‐compatible RF applicator for mild RF‐HT. Using the PDF and the LBV algorithm, we correct for the susceptibility artifacts in a simulated case, during a phantom heating experiment, in volunteer data, and for MR thermometry data during mild RF‐HT of cervical cancer.

## 2 THEORY

### 2.1 Superposition of different fields

During digestion, feces and gases move through the colon, creating field alterations that contribute to the phase difference image between 2 time points.

This change in the magnetic field can be related to the change of susceptibility via convolution with a dipole‐kernel in k‐space. The change in the relative difference field with respect to the nominal B_0_ field ( ΔRDF) can be related to a difference in the susceptibility distribution ∆χ and is expressed in image space per^32^:

(1) $$\Delta RDF\left(r\right)=\frac{\Delta \left[B\left(r\right)-B_{e}\left(r\right)\right]}{B_{0}}=\frac{1}{4\pi }\int \Delta \chi \left(r^{\prime }\right)\frac{\left(3cos^{2}\theta -1\right)}{{\left|r^{\prime }-r\right|}^{3}}d^{3}r^{\prime }$$

The relative field change relates the change of the difference between total induction field $B\left(r\right)$ and the external background magnetic field $B_{e}\left(r\right)$ to $B_{0}$. The angle θ denotes the angle between $r^{\prime }-r$ and the unit vector in z‐direction.

In our case, the phase image used is the phase difference image from 2 time points, and the “background field,” in fact, is the field change‐induced by a different susceptibility distribution due to intestinal motion of gasses.

## 3 METHODS

All measurements were conducted on a 1.5T GE system (GE Discovery MR450w General Electric, Milwaukee, Wisconsin). The BSD2000‐3D Sigma Eye MR‐compatible RF applicator (PYREXAR Medical, Salt Lake City, Utah) consists of 24 dipole antennas arranged in 3 rings of 8 antennas (Figure 1A). The applicator operated at 100 MHz simultaneously to the MR image acquisition. Coaxial filters prevented the RF waves from interfering with the MRI signal acquisition. The water bolus between the antennas and the patient was cooling the patient by circulating water during the treatment. No water was circulated during the phantom measurement or the volunteer study. RF‐immune thermistors with high‐impedance carbon wires (“Bowman” temperature probes^33^) were guided into catheters to monitor the temperature locally. Only the body coil was used for signal reception because coils between the applicator and the patient body would interfere with the RF heating. Simulations, image reconstruction, and processing were done in MATLAB (MathWorks, Natick, Massachusetts). For PDF and LBV, the MATLAB scripts from the MEDI toolbox were used (http://pre.weill.cornell.edu/mri/pages/qsm.html). The tolerance value was set to 0.01 for the PDF method. Default values for the LBV method were used (tolerance = 0.01, depth = −1, peel = 0, N1/N2/N3 = 30/100/100) for all in vivo data sets and the phantom experiment. For the simulation, we set depth = 1 and peel = 3. For phase unwrapping, we used the code available at https://gitlab.com/veronique_fortier/Quality_guided_unwrapping.^23^ Four patient scans were conducted with the approval of the local ethics board.

### 3.1 Determination of motion source

Motion within the pelvis may originate from muscle contraction and relaxation, involuntary bulk motion, breathing, and digestion. To disentangle the potential motion sources during mild HT, we observed the phase evolution with a high temporal resolution within a single slice of the pelvis of 4 volunteers over time, within and outside a filled water bolus. In addition, the recorded signal of a respiratory belt was compared to the phase signal evolution within a ROI inside the pelvis. A single‐slice multiphase Fast‐SPGR (standard spoiled gradient echo) sequence was used with the following scan parameters: echo time (TE) = 5 ms, pulse repetition time (TR) = 7.22 ms, matrix size = 128 × 46, field of view (FOV) = 50 cm × 50 cm, reduced phase FOV = 0.7, slice thickness = 10 mm, scan time per phase = 694 ms. For better phase sensitivity and larger coverage of the FOV, we used slightly different scan parameters for the scans including the applicator and water bolus: TE = 10 ms, TR = 12.3 ms, slice thickness = 8 mm, matrix size = 128 × 64, FOV = 50 cm × 50 cm, bandwidth = 31.25 kHz, scan time per phase = 837 ms. Acquiring only single echo was sufficient because there is no conductivity bias at constant temperature, and therefore, no need to correct for it via a double echo acquisition scheme.

### 3.2 Simulation

The impact of moving gas within the intestines on the magnetic field and possible correction methods were first studied via field simulations.

The change in the magnetic field caused by a distribution of susceptibility differences can be simulated via the convolution of the susceptibility change with a magnetic dipole in the Fourier domain.^34^ The simulated matrix size was 150 × 150 × 150, which we corresponded to a FOV of 50 × 50 × 50 (cm)^3^. In a first step, we simulated the field disturbance originating from a susceptibility change Δχ within a sphere with a diameter of 2 cm in the center of the image. The susceptibility difference Δχ was the one between water and air. A 3D Gaussian temperature increase profile with a peak value of 10°C and a standard deviation of 5 pixels was added to the image. In a second scenario, a spatially linear B_0_ field drift was added to the susceptibility change and the temperature rise. PDF and LBV were applied in both cases to remove the background fields by defining a central sphere of 2 cm diameter as the background mask and the remaining image as the foreground.

### 3.3 Phantom heating experiment

To validate the BFR performance quantitatively we reprocessed previously acquired data from a phantom heating experiment. A DEGRE with slice interleaved acquisition scheme was scanned for temperature monitoring. Using the phase signal at both TEs compensated for conductivity change‐induced phase offsets.^7^ Scan parameters were TR = 620 ms, 25 slices, total scan time = 83 s, TE_1_ = 4.8 ms, TE_2_ = 19.1 ms, matrix size = 128 × 128, FOV = 50 cm × 50 cm, flip angle = 40°, slice thickness = 10 mm, bandwidth = 325.5 Hz/px.

Our results were compared with temperature probes and the previous B_0_ drift corrected DEGRE MR thermometry method. The cylindrical phantom mimicked the electrical properties of human tissue. It was made of 89.76% demineralized water, 10% of a hydrophilic organic polymer solidifying powder, called “super stuff” (TX‐151), 0.205% NaCl, and 0.04% NaN3 (percentage values of the total weight are given), and was used in previous validation experiments.^26^ The phantom was 30 cm in diameter and 40 cm in depth. Several catheters passed through the phantom parallel to the cylinder’s axis, allowing for the insertion of temperature probes. After the acquisition of the baseline reference images at a scanner room temperature of 20°C before heating, the BSD applicator was turned on at 400 W with phase settings to achieve a focus 3 cm below the center of the applicator. DEGRE scans were acquired. The RF power was turned off after 25 minutes of heating.

For the reference DEGRE scan and for the current DEGRE image, the phase difference between both echoes was calculated, respectively, via complex multiplication. The resulting difference phase images were again subtracted from each other. The final conductivity bias‐corrected phase difference image was unwrapped. For defining the background and foreground masks, the short echo time images were used because of their higher SNR. Because the BFR algorithms subtract 0th and 1st order spatial phase variations, not only the B_0_ drift, but a potential heating following this spatial phase pattern would be subtracted from the final phase difference in case of the phantom heating experiment. Applying following steps would account for spatially linear heating: A constant and linear phase contribution $\Delta \phi_{0th+1st}$ was fitted within the ROI. This was added to the BFR corrected phase, which removed both dipolar phases as well as phases of 0th and 1st order. A 0th and 1st order B_0_ drift contribution $\Delta \phi_{\mathrm{Drift}}$ was fitted using only the reference tube signals (Figure 1B). This phase was subsequently subtracted from the phase obtained after either LBV or PDF correction $\Delta \phi_{\mathrm{BFR}}$ to obtain $\Delta \phi_{\mathrm{final}}$ (Supporting Information Figure S1).

(2) $$\Delta \phi_{\mathrm{final}}=\Delta \phi_{\mathrm{BFR}}+\Delta \phi_{0th+1st}-\Delta \phi_{\mathrm{Drift}}$$

**FIGURE 1:**
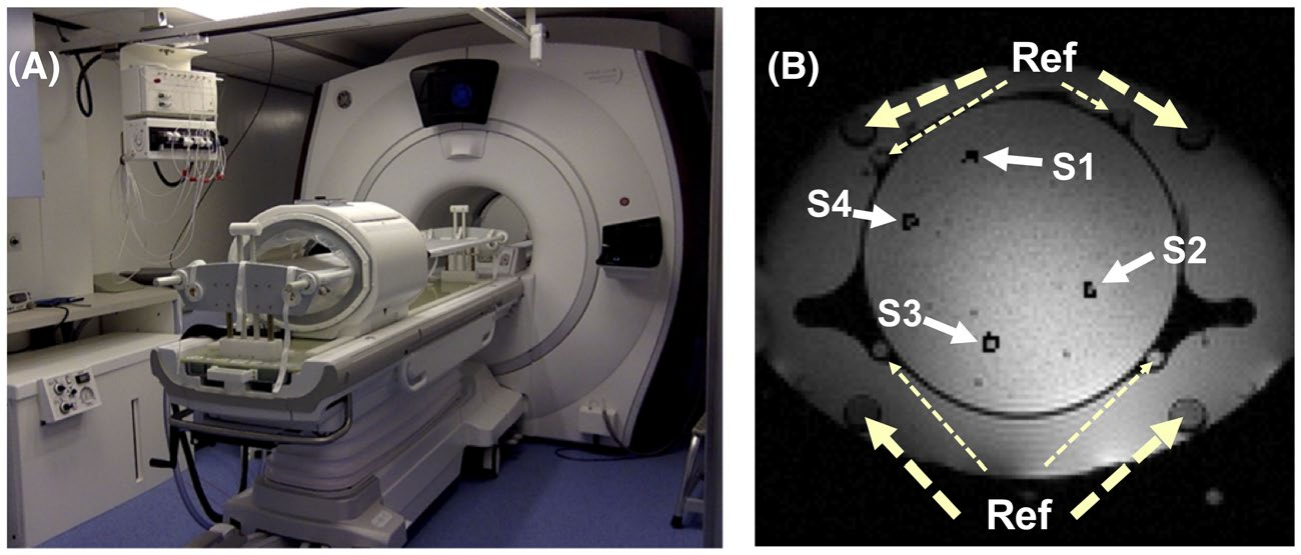
A, The used hybrid system for MR‐monitored mild radio frequency‐hyperthermia (RF‐HT) consisted of a 1.5 T MR scanner and a MR‐compatible RF applicator. The water bolus within the RF applicator is connected via plastic pipes to the water container visible on the bottom right of the image. B, Axial slice showing the phantom heating setup. S1 to S4 display the location of the image regions of interest (ROIs) around the sensor tip location within respective catheters that served for the evaluation of the heating profile. “Ref” points at the larger 4 reference tubes containing silicone as well as additional smaller reference tubes filled with peanut oil

### 3.4 Stability study in volunteers

To prove the feasibility of the proposed method, we conducted a substudy on volunteers at constant temperature. Four volunteers (3 male and 1 female) were scanned in the pelvis using a spoiled single‐echo gradient echo acquisition scheme at constant temperature (TE/TR = 15 ms/21 ms, 20 slices matrix size = 128 × 160, FOV = 50 cm × 50 cm, flip angle = 14°, slice thickness = 10 mm). The second scan was acquired 30 minutes after the reference scan. A threshold was defined as 60% of the mean magnitude value over the data set and was applied to generate tissue masks for each time point. The overlapping area of the mask at the current time point and the reference time point was defined as our foreground mask that is needed for the BFR. LBV or PDF was then applied using the unwrapped phase difference image and the computed mask to obtain the remaining phase difference that is free of susceptibility error and B_0_ drift. The post‐processing pipeline corresponded to the flow chart presented in Figure 2 and was used for both volunteer and patient treatment data.

**FIGURE 2:**
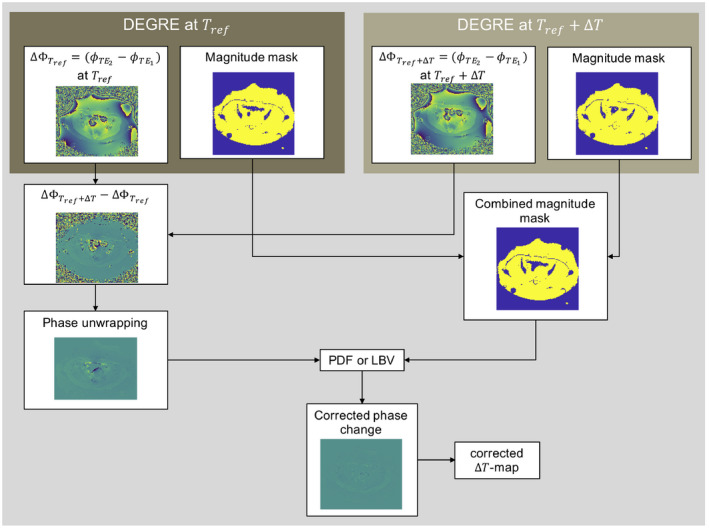
Flow chart describing the pipeline for susceptibility artifact correction via background field removal algorithms using Double Echo Gradient Echo (DEGRE) data. For conductivity bias correction, the phase difference of long and short echo images is computed for the reference and current time point. The phase difference image of both phase differences usually needs to be unwrapped because large field gradients are introduced in the presence of susceptibility artifacts. A combined magnitude mask from both data sets based on the short echo time image is computed with the logical AND‐operator. Applying the projection onto dipole fields (PDF) or Laplacian boundary value (LBV) algorithm removes the linear B_0_ drift and the susceptibility artifacts and ideally only preserves the temperature‐induced field change for generating the final temperature increase map

### 3.5 In vivo DEGRE data during mild RF‐HT treatment

Four patients with cervical cancer were treated with deep mild RF‐HT with a treatment duration of 90 minutes. Treatment planning causing a heated focus within the cervical tumor was done using Sim4Life (Zurich MedTech AG, Zurich, Switzerland) based on the patient’s anatomical model segmented from a computed tomography data set (Supporting Information Figure S2). MR thermometry scans were acquired every 10 minutes. The scan parameters for the DEGRE acquisition were the same as described for the phantom heating experiment. The Bowman temperature probes were inserted into natural lumen, to the uterus via the vagina and the rectum through catheters. The temperature in the perineum was also measured via a temperature probe. The same post‐processing steps as for the volunteer data were applied.

## 4 RESULTS

### 4.1 Determination of motion source

The results within a male volunteer are illustrated in Figure 3. The first row shows the results without the RF applicator around the pelvis. Respiratory motion‐induced phase shifts would be misinterpreted as temperature changes of up to 7°C peak to peak in the studied ROI if not corrected for. The periodic field fluctuations disappeared when placing the subject within the applicator and filling the water bolus (Figure 3B‐2). This might be partly due to restricted respiratory motion by mechanical pressure from the water bolus, but it is mainly due to the absent temporal change of the susceptibility distribution within the magnetic field in the presence of the water bolus with breathing.

**FIGURE 3:**
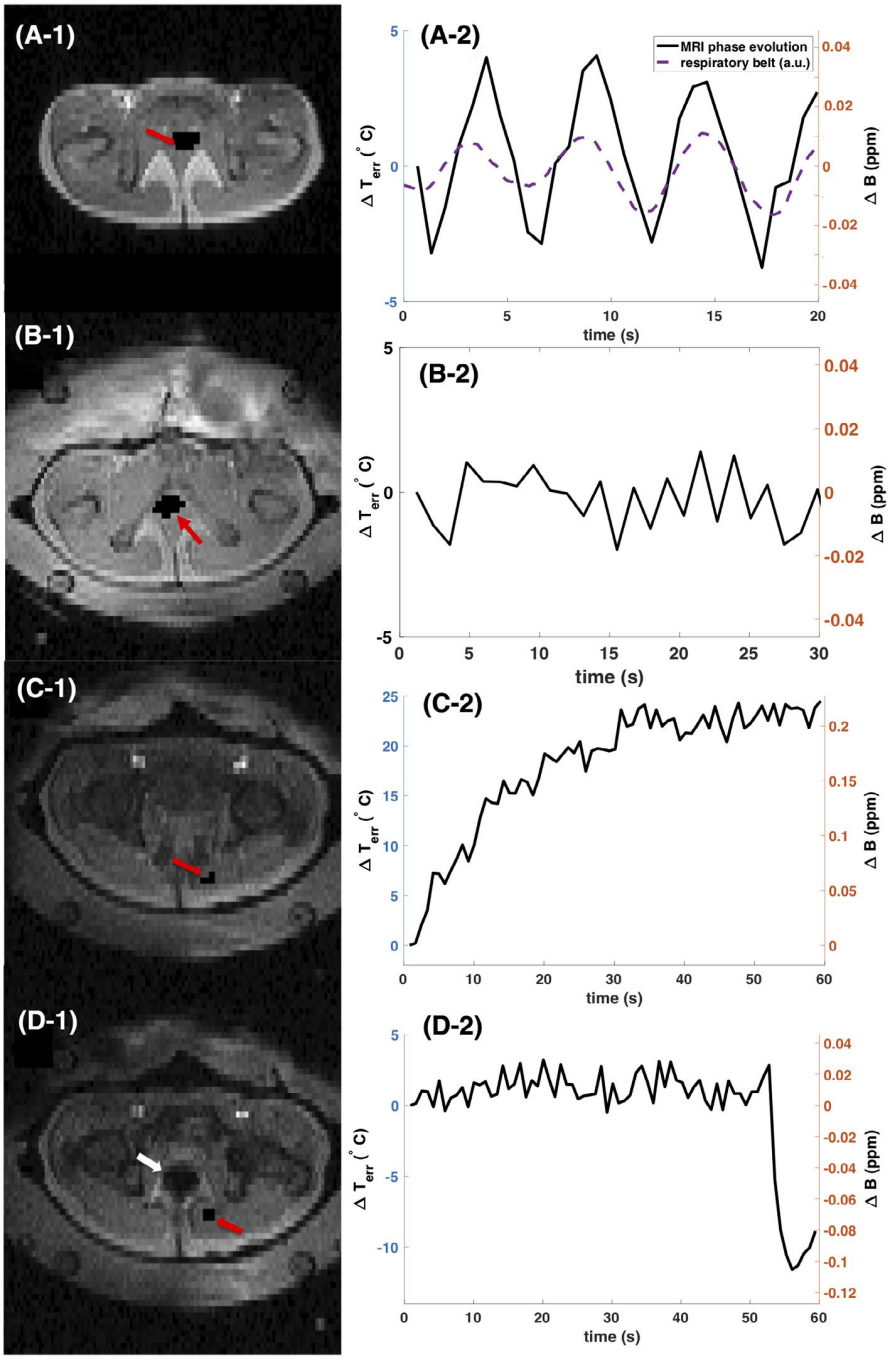
Disentangling motion‐induced susceptibility artifacts during proton resonance frequency shift (PRFS) MR thermometry in the pelvis outside and inside the RF‐HT applicator. The left column displays the magnitude data of the studied 2D MR slice of the pelvis. Red arrows point at the region of interest (ROI) for which the mean phase evolution in ppm and the resulting erroneous temperature estimation over time are plotted in the right column. The white arrow points at digestive gas appearing in the imaged slice. Even though no tissue displacement was visible, except for the intestinal gas induced tissue deformation in image D‐1, significant phase variation was observed in all cases due to the propagating character of the dipole‐shaped susceptibility artifacts. In the first row the subject was placed inside the scanner bore without the applicator. In the second, third, and fourth row the RF‐HT applicator was positioned around the pelvis and the water bolus was filled with water

Also for panels C and D in Figure 3, no breathing‐induced field fluctuation was observed when the RF applicator was surrounding the pelvis. Instead, a continuous field drift was detected within the ROI displayed in Figure 3C, which corresponded to an erroneous temperature increase of nearly 25°C. However, no motion was observed in the magnitude data in that slice. In contrast, a sudden field change (Figure 3D) of about 0.1 ppm was observed. The results illustrate that digestive motion is irregular and introduces major temperature estimation errors in regions far from the origin of motion.

### 4.2 Simulation

The results from the simulation study are plotted in Figure 4. Both PDF and LBV could remove the dipole field caused by a spherical susceptibility change while preserving the simulated Gaussian distributed temperature increase. In case no additional linear B_0_ drift was applied, both methods performed similarly well. At the image border, we observe a slight offset for the PDF corrected data of less than 0.1°C. As we can observe in Figure 4B‐2 and the line plot in Figure 4E‐2, PDF does not resolve the linear B_0_ drift as well as LBV does. This could be attributed to the fact that PDF only projects dipole sources within the FOV. Furthermore, the error distribution plot in Figure 5 suggests a higher precision for the LBV method for the simulated data with B_0_ drift correction. The 5th to 95th percentile interval of the calculated temperature error amounted to 0.26°C for PDF corrected data and 0.009°C for LBV.

**FIGURE 4:**
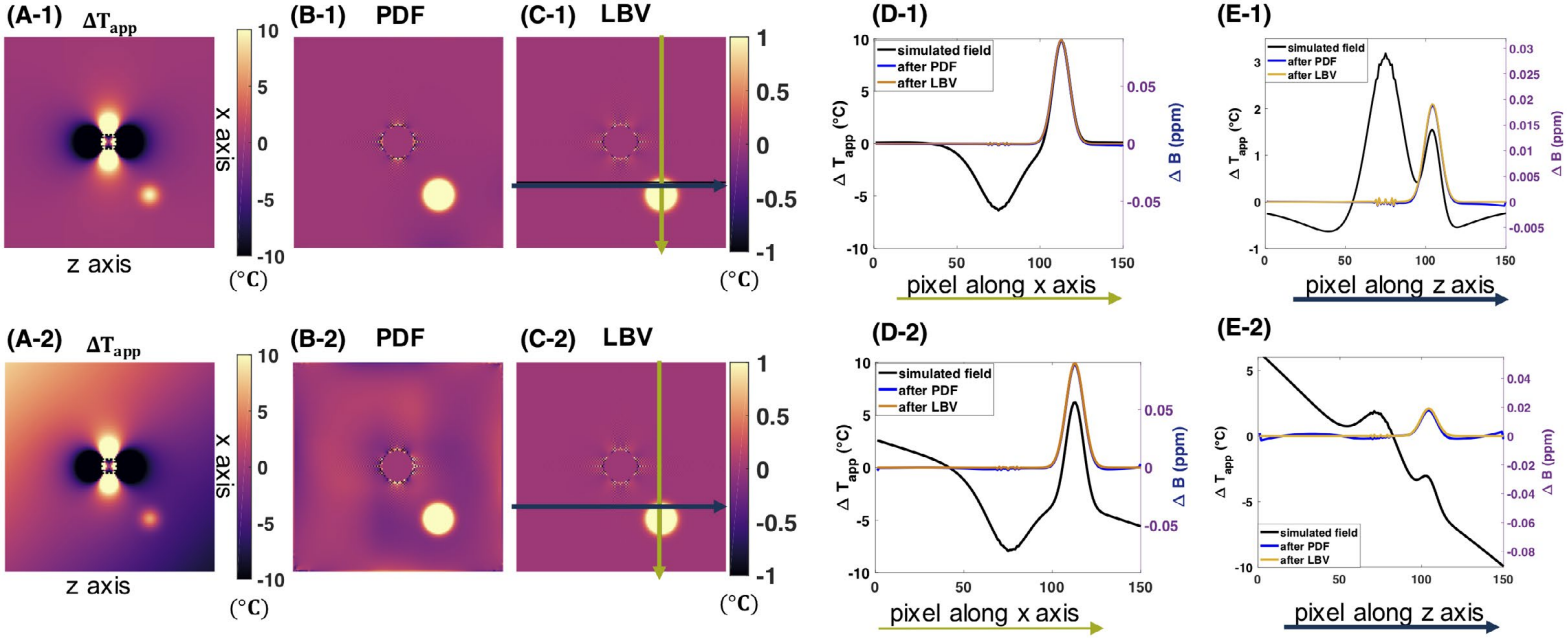
Simulation results of field disturbance introduced by susceptibility change and the impact of background field removal. The first row represents a case where a susceptibility change in the center of the image caused a dipole field in the phase difference image. In addition, a Gaussian distributed temperature rise was added. In the second row, a linear B_0_ drift was added on top of the temperature rise and susceptibility change‐induced field changes. A, apparent temperature change in °C if no correction was applied, B, apparent temperature increase after projection onto dipole fields (PDF), C, apparent temperature increase after Laplacian boundary value (LBV), D, line profile of the simulated original field and the PDF‐ and LBV‐corrected fields and apparent temperature change, corresponding to the vertical green line illustrated in C), E, line profile of the simulated field and the PDF‐ and LBV‐corrected apparent temperature and field, corresponding to the horizontal black line illustrated in C). Z axis corresponds to the orientation of the main magnetic field, whereas x axis is perpendicular to it. The nonsmooth, noise‐like field variation in the middle of the axis originates from numerical simulation of the disturbing field from a nonperfect sphere and persists after background field removal (BFR) correction. However, this is not a concern for experimental or in vivo data

**FIGURE 5:**
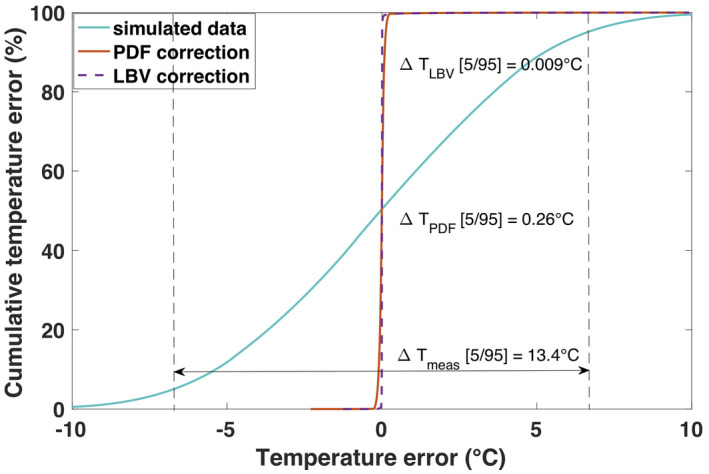
Cumulative distribution functions of the temperature error before (turquoise line) and after application of the background field removal methods, projection onto dipole fields (PDF) or Laplacian boundary value (LBV). The simulation data with B_0_ drift, as illustrated in Figure 4‐2), is represented. The entire simulated 3D volume was used for calculation of the cumulative error

### 4.3 Phantom heating experiment

Representative temperature change maps ( ΔT‐maps) using different correction methods are plotted in Figure 6. When comparing the drift‐corrected ΔT‐maps with the LBV‐corrected ΔT‐maps (Figure 6E‐1) we see what is found as a background field by the LBV algorithm. Even though we did not anticipate any motion‐induced susceptibility artifacts in the phantom and we could not see any motion in the magnitude data, we can still observe small dipoles appearing at the location of the catheters. These results indicate a subvoxel displacement of the phantom, which caused the dipole fields around the air‐filled catheters. In case of the slice at the border of the FOV, we observe a larger dipole in Figures 6A‐2 and B‐2. This dipole field, which was likely caused by a moving air bubble in the surrounding water bolus, causing local temperature errors of up to ±8°C, was removed by both BFR algorithms. This artifact was also penetrating visibly to the neighboring 4 slices and still caused an offset of about 1°C in slices that were further away. The white arrows in Figure 6 point at border regions in the ΔT‐map, where a slight temperature decrease was detected, and is due to overfitting of the background field by PDF.

**FIGURE 6:**
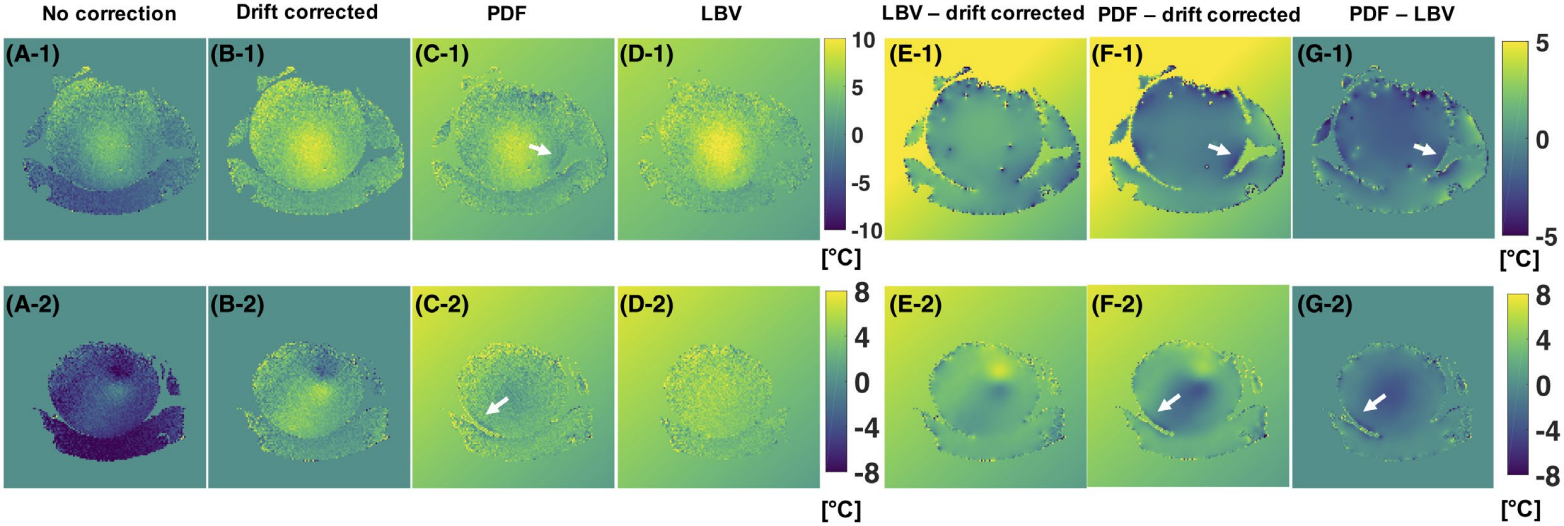
Temperature change maps ( ΔT‐maps) from the phantom heating experiment using Double Echo Gradient Echo (DEGRE) data acquisition and different correction methods for 2 different slices. The last time point, 50 minutes after the acquisition of the reference image, was chosen. The first row displays the temperature increase maps for a middle slice. The second row displays the temperature change maps at the border of the phantom, in which we observed the formation of a dipole field from some motion‐induced susceptibility change. A, ΔT‐maps derived from DEGRE data without correcting for B_0_ drift, B, ΔT‐maps with B_0_ drift correction, C, ΔT‐maps after applying PDF for background field removal (BFR), D, ΔT‐maps after applying Laplacian boundary value (LBV) for BFR, E, difference of ΔT‐maps using the method shown in B) and D), F, difference of ΔT‐maps using projection onto dipole fields (PDF) and only drift correction, G, difference of ΔT‐maps PDF compared to LBV method. The white arrows point at areas where PDF overestimated the background field locally at the border of the phantom

Figure 7 compares the mean temperature values of the different correction methods with the temperature probe data for the entire duration of the phantom heating experiment. The sensor locations and the respective ROIs for which the mean and standard deviation values are calculated are illustrated in Figure 1B. The mean absolute deviation from the temperature probe data over all 44 data points is 0.678°C for DEGRE, 0.752°C for the LBV, and 0.873°C for the PDF method. The accumulated standard deviation across the ROIs for all 44 measurement points were 16.13°C for DEGRE, 14.28°C for the LBV‐corrected ΔT‐maps, and 15.95 for the PDF‐corrected ΔT‐maps. Because of uncertainties regarding the exact probe positioning and because all methods matched the probe data quite well, it was not possible to judge on the accuracy of the different methods at the location of the probes. However, the smaller standard deviation values for the ΔT‐maps with BFR suggest, in accordance with Figure 6E‐1, that we could get rid of local phase variations due to the small dipoles created by a subvoxel displacement of the phantom.

**FIGURE 7:**
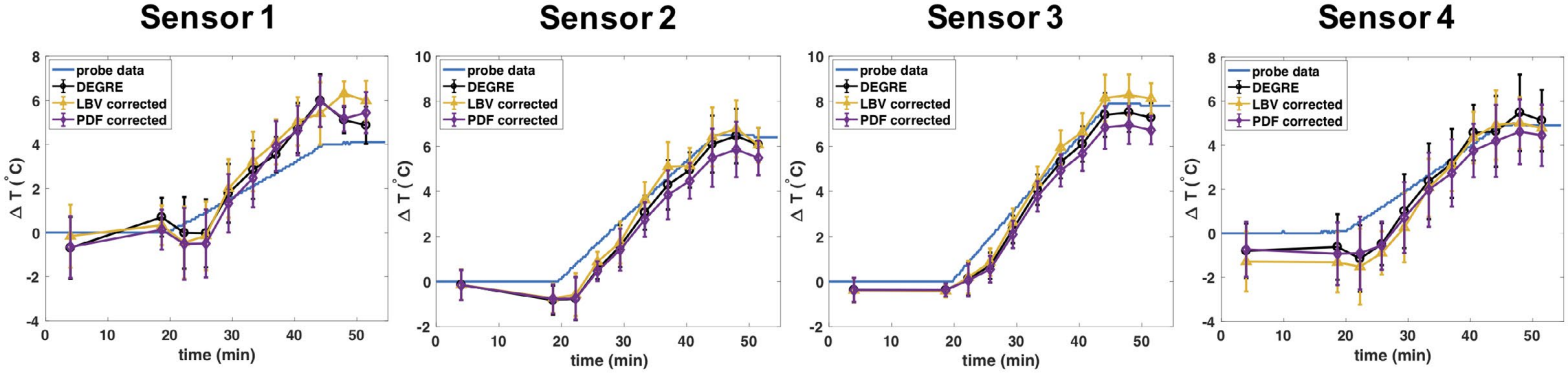
Temporal evolution of mean and standard deviation of temperature change values within small regions of interest (ROIs) surrounding the temperature probe location (as indicated in Figure 1) over the course of the phantom heating experiment. Local temperature change values obtained from B_0_ drift corrected Double Echo Gradient Echo (DEGRE) maps are compared to results obtained with projection onto dipole fields (PDF) and Laplacian boundary value (LBV) as well as to the probe data

### 4.4 Stability study in volunteers

In all 4 volunteers, severe digestive motion‐induced susceptibility artifacts could be observed within 30 minutes after the reference scan (Figure 8). Because no bulk motion occurred, applying the BFR methods could eliminate the field disturbance down to noise level. In subject 1 and 2, no remarkable difference between PDF and LBV could be observed. In subject 3 and 4, LBV removed the background field more robustly, which can be seen by the larger interval between the 5th and 95th percentile in the cumulative temperature error plots, as well as the residual dipoles, that white arrows point at in Figure 8.

**FIGURE 8:**
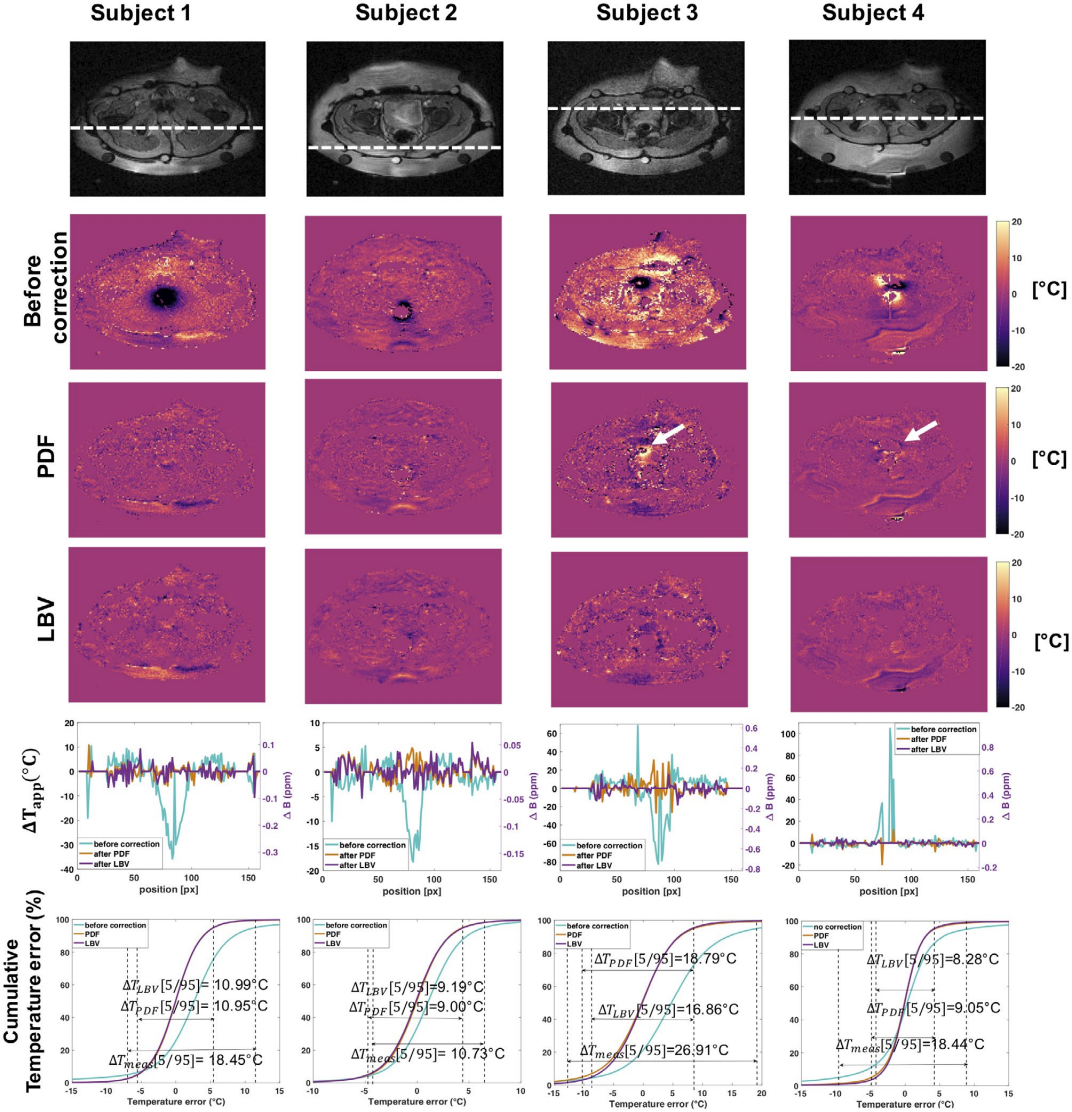
Results of stability study in 4 healthy volunteers at constant temperature. 1st row: magnitude image of the slice for which the temperature maps are displayed below. The dashed line corresponds to the location of the line plot in the 5th row of the same figure. 2nd row: temperature error maps due to intestinal motion containing gas. 3rd row: temperature error maps after projection onto dipole fields (PDF) correction. The white arrows point at locations where large residual phase is still observable after background field removal (BFR). 4th row: temperature error maps after Laplacian boundary value (LBV) correction. 5th row: line plots displaying temperature errors before and after BFR correction in the respective subject at the location shown in the first row. 6th row: cumulative distribution functions of the temperature error before and after BFR. Only voxels within the subject were considered, excluding pure noise locations and data from the water bolus

### 4.5 In vivo DEGRE data during mild RF‐HT treatment

Extracts from corrected temperature increase maps during 1 patient’s treatment are displayed in Figure 9. While for the first row no heating was applied yet, the second row displays a slice after 30 minutes of heating. Severe intestinal gas motion‐induced susceptibility artifacts affected each slice within the scanned pelvis and even spread to the water bolus. The linear B_0_ drift could be eliminated completely via background field removal (Figure 9E‐1), and the susceptibility artifacts reduced significantly. Areas close to the gas/tissue interface are likely to keep showing a wrong temperature change after correction. The 5th to 95th percentile interval of the temperature error distribution decreased from 17.14°C to 9.81°C after PDF correction and 9.56°C after applying LBV (Figure 9F) for the temperature map before heating. Due to the intrinsically low SNR, phase noise restricts the precision of the corrected temperature maps. Nevertheless, a heated area with a width of about 6 cm width could be detected after correction (red arrow in Figure 9E‐2), which was misinterpreted as −25°C before BFR. The temperature after PDF correction seems overestimated at the target region, because temperature difference values of above 10°C were detected in the PDF‐corrected line profile, but a maximum temperature of only about 43°C is expected. At the location of the black arrow (in Figure 9E‐2), the PDF‐corrected line profile suggested a slight cooling of the tissue, which is also not physiological. In this line profile, the LBV method yielded more realistic values at the arrow locations.

**FIGURE 9:**
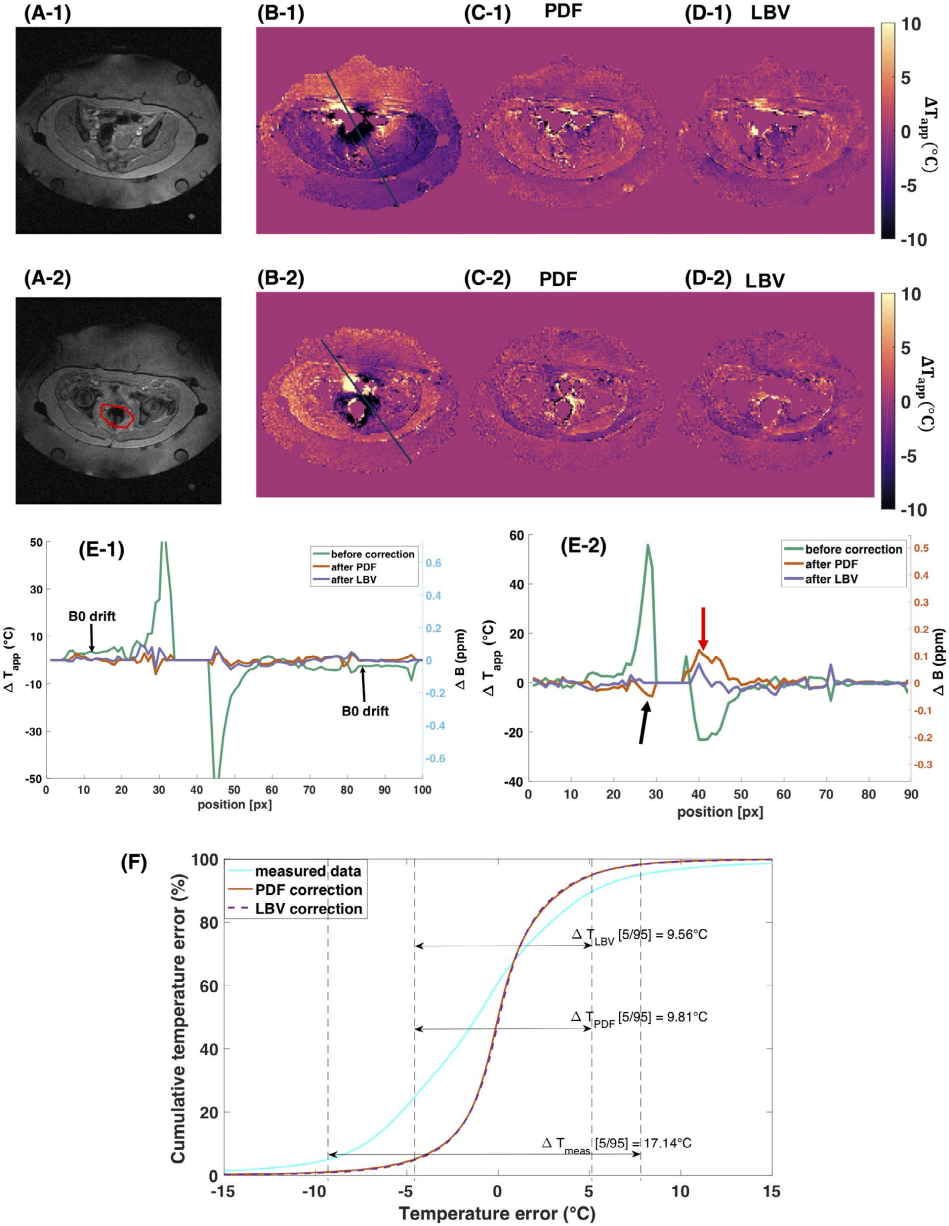
Results of susceptibility artifact and B_0_ drift correction in Double Echo Gradient Echo (DEGRE) data during mild RF‐HT of the pelvis. The first row represents the temperature difference maps for 2 time points with different susceptibility distributions before heating. The second row represents the temperature difference maps with heating. A, magnitude image of the slice for which the temperature maps and profiles were plotted, respectively. B, uncorrected apparent temperature increase maps. C,D, temperature increase maps after projection onto dipole fields (PDF) or Laplacian boundary value (LBV), respectively. E, line profiles for the temperature maps displayed in B‐D). The red arrow in E‐2) points at a location with heating that was recovered by both background field removal (BFR) methods. The black arrow in E‐2) points at a location where the PDF method slightly underestimates the temperature. The red line in A‐2) delineates the target area and was extracted from the computed tomography data based treatment planning (Supporting Information Figure S3). It is important to note that the spreading character of the susceptibility artifact made it impossible to use the signal in the reference tubes for B_0_ drift correction in the in vivo scenario. F, represents the cumulative error of the DEGRE data set before applied heating, as illustrated in Figure 9‐1). It was calculated based on the image mask excluding areas of noise

Out of the 4 patient treatment data sets we examined, one showed severe bulk motion, where the entire pelvis was displaced between scans. A second data set contained too much gas, that little tissue was visible after BFR correction. Therefore, we could only robustly apply BFR on 2 treatment data sets, but for their entire treatment duration of 100 minutes and 70 minutes in the second case (Figure 10).

**FIGURE 10:**
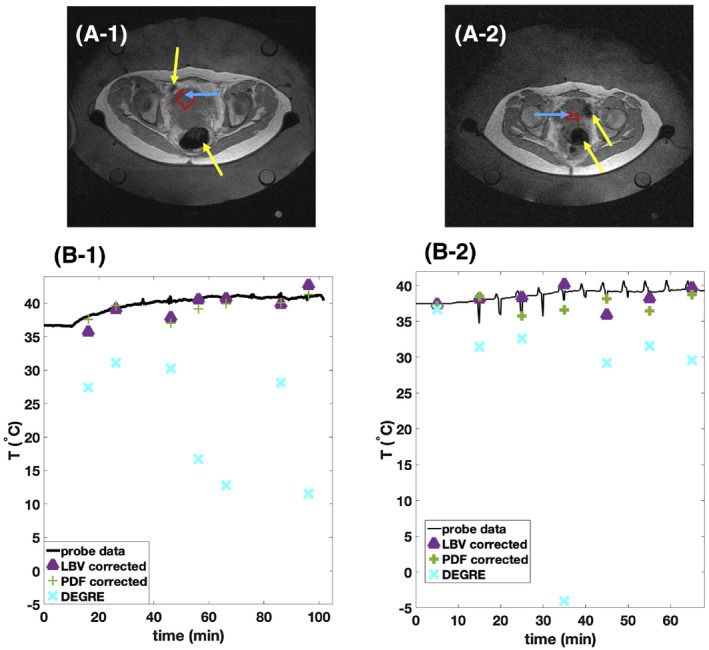
Comparison of background field removal (BFR)‐corrected MR thermometry to Double Echo Gradient Echo (DEGRE) data and temperature probe data for 2 treatments of RF‐HT of cervical cancer. A, High resolution scan done previously to heating displays the cervical tumor within the pelvis. Yellow arrows point at intestinal gas. Blue arrows point at the catheter’s location visible as a tiny dark spot that can be tracked through the slices. Red lines show the borders of the considered region of interest (ROI) for evaluation of MR thermometry data. B, The mean MR thermometry values within the shown ROI in the tumor obtained from DEGRE data and BFR corrected data is compared to the probe data. The temperature change values obtained from MR data were added to the initial baseline temperature of the temperature probe

We have plotted the mean MR thermometry values within a ROI around the location of the temperature sensor tip within the tumor (blue arrows in Figure 10A) together with the probe data in Figure 10B. As seen in Figure 10B, the DEGRE data with only B_0_ drift correction show no useful temperature information due to motion‐induced susceptibility artifacts. Figure 10B‐2 shows better matching of the LBV‐corrected data with the temperature probe compared to the PDF‐corrected data, whereas LBV and PDF performed similarly in Figure 10B‐1. However, at certain measurement time points, a deviation up to 3°C was observed for both methods, which could be due to intrascan motion.

## 5 DISCUSSION

The main susceptibility artifact contribution during PRFS‐MR thermometry within the pelvis originates from intestinal gas motion because the water bolus eliminates respiratory motion‐induced field changes. Figure 3 suggests that the field change due to digestion can be gradual or sudden. Because the requirement for the temporal resolution during mild RF‐HT is only in the order of 10 minutes, the slice interleaved DEGRE acquisition scheme with 83 s scan duration for the data collection of 25 slices is acceptable, since the main phase contrast contribution will come from the acquisition time point of the central k‐space lines. As seen from the phantom heating experiment, the BFR algorithms can correct for the field changes between 2 scans, if the fields are correctly represented. As seen in the in vivo data sets it is less powerful in case the intestinal gas moved during the scan, because intrascan motion artifacts affected the image phase. To avoid the effect of intrascan motion effects on the correct phase information, usually a high temporal resolution as of less than a second per image frame would be needed, which is not feasible with a 3D volume coverage. However, a big volume coverage is desired for a robust estimation of the disturbing “background field.” As shown in Figure 3B, there are extended periods of time without motion influences on the phase signal. Given a low required temporal resolution of temperature maps for monitoring of mild RF‐HT of about 10 minutes, one could think of interrupting and repeating the scan whenever intrascan motion is detected or constantly acquiring data and subsequently discarding images with intrascan motion. The latter solution may raise concerns about patient comfort due to acoustic noise. A more time‐efficient acquisition scheme, for instance using a fast spin echo sequence^26^ or a slightly reduced FOV would increase the likelihood of scanning during a time period without intrascan motion. Although applying BFR worked robustly on the volunteer data sets, interscan bulk motion due to patient discomfort is more likely to occur during mild RF‐HT and was the reason of the dropout of 1 examined patient treatment data set. To account for bulk motion, a previous work has proposed to use later scans as a new reference and register the temperature difference maps with regard to each other.^35^

Both PDF and LBV remove a spatially linear and constant phase change. In case of the phantom heating experiment, we could differentiate the temperature‐related linear and constant phase from the B_0_ drift contribution by fitting a constant and first‐order phase distribution once over the image volume and once only over the reference tube signal. In case of the in vivo data, we did not expect a linear temperature rise because of the applied cooling. Furthermore, the spatial extension and large magnitude of the susceptibility artifacts affected the reference tube signal severely that it was not possible to extract the B_0_ drift from the reference tubes. Moreover, the very low SNR in the reference tubes hamper B_0_ drift calculation. Both PDF and LBV overcome this problem and remove the linear B_0_ drift effect robustly based on the data in the entire ROI.

BFR methods can represent a very elegant way to remove the constant and linear components of the B_0_ drift also for other thermal treatments, where the heating pattern is locally confined, such as for MRgHIFU. No additional hardware, such as reference tubes and no additional signal acquisition, such as proposed recently using additional free induction decay signals from different coils,^30^ would be needed. BFR algorithms could also elegantly correct for temperature change‐induced susceptibility artifacts^36^ and replace the forward‐calculation of the disturbance field and additional data acquisition such as T1 maps in fat.^12^

The location of the tissue‐gas boundary areas within the pelvis are especially challenging because the targeted tumor is oftentimes located near these boundaries. It inherently suffers from a low SNR because of the strong magnetic field gradients at tissue‐gas interfaces. In addition, the LBV method removes 1 more pixel layer compared to the PDF method to solve the boundary formulation. The PDF method may be less sensitive to noisy boundary data but is known to have problems resolving the local field at the tissue border correctly,^18^ which could be observed in the phantom heating data in Figure 6.

Sufficient SNR and thus temperature‐to‐noise ratio is important for the desired precision for monitoring mild HT. The current slice thickness is 1 cm and the in‐plane voxel size is 3.9 mm in both x and y direction. The low spatial resolution limits the correct representation of the susceptibility‐induced field change and, therefore, the power of the background field removal step. Using a 3D gradient echo acquisition scheme, a fast spin echo sequence,^26^ or switching to 3 T, would all potentially lead to higher SNR. Enough image coverage is needed to detect potential hotspots and is advantageous for reliably solving the background field problem but requires longer scan time. Future work should accommodate the trade‐off between temporal and spatial resolution, volume coverage as well as an improved temperature‐to‐noise ratio.

Previous work employing PDF in the context of PRFS‐based MR thermometry was limited to a 2D slice to correct for motion‐induced susceptibility artifacts at constant temperature in humans as well as during MRgHIFU in a pig.^16^, ^17^ The anatomical target region was the abdomen, which is much more affected by respiratory motion than the herein investigated pelvic region. Even though the authors of the paper have noticed superiority of a 3D implementation of PDF over the 2D correction in an ex vivo tissue sample, the authors decided to continue with only the 2D implementation due to the requirement of a high temporal resolution at the presence of breathing‐induced tissue displacement and field variations. In contrast, only a low temporal resolution is required for monitoring mild RF‐HT in the pelvis. We can therefore profit from a 3D coverage if we deal with the potential phase errors due to intrascan motion.

Instead of applying the BFR after the phase subtraction step between 2 different time points (and thus temperatures), we checked the performance of both BFR algorithms when applied for each time point separately, as a step before subtracting the phases between 2 temperature distributions. We compared the 2 approaches for both BFR algorithms by applying them to the phantom heating experiment (Supporting Information Figure S2). The PDF algorithm, that minimizes the field distribution within a chosen ROI detected the temperature increase falsely as background field in certain cases (Supporting Information Figure S2:A2+A4). In contrast, LBV overestimated the background field contribution only locally when applied as a step before phase subtraction (Supporting Information Figure S2:B6+B10). However, both BFR methods yielded more robust results when applied only once.

Medication or the use of a preparatory micro‐enema^37^ could slow down or inhibit digestive motion temporarily. The insertion of a liquid rectal filling has also shown an artifact reduction.^38^ However, reduced patient comfort and possible side effects are oftentimes a strong contraindication.

Even though the motivation for our work was related to mild RF‐HT inside the pelvis, the potential area of application of the BFR techniques for PRFS‐based MR thermometry is not limited to this anatomical area, nor on the mild heating range of the treatment.

## 6 CONCLUSION

Field perturbations due to breathing were negligible for mild regional RF‐HT within the pelvis in the presence of the surrounding large water bolus of the RF applicator. The severity of digestive gas motion‐induced susceptibility artifacts on PRFS‐based MR thermometry with its spatial extensiveness and temperature error amplitude was shown.

The LBV and PDF methods improved the MR thermometry data by significantly reducing the susceptibility‐induced temperature error:

Applying the BFR methods could remove susceptibility artifacts during a phantom heating experiment without compromising accuracy and precision compared to the noncorrected data. The stability of the method was shown in 4 volunteers, where bulk motion could be excluded. PDF and LBV‐corrected DEGRE data matched temperature increase values measured with reference temperature probes for 2 patient data sets during mild regional RF‐HT of cervical cancer.

In comparison to LBV, PDF showed a tendency to overestimate the background field contribution at borders of the ROI, which could be observed in the simulated data, the phantom heating data and the volunteer data sets. LBV performed more robustly but has the disadvantage of removing 1 pixel layer for calculating the disturbing field.

B_0_ drift correction comes for free without the need for additional measurements or reference tubes.

## CONFLICT OF INTEREST

Marion I. Menzel is employed by GE Healthcare.

## Supporting information


**FIGURE S1** Flow chart describing the pipeline for susceptibility artifact correction via background field removal algorithms using Double Echo Gradient Echo (DEGRE) data in case for the phantom heating data. For conductivity bias correction, the phase difference of long and short echo images is computed for the reference and current time point. The background field removal (BFR) is applied on the phase difference image of both phase differences, and removes susceptibility artifacts but also any spatially linear or constant phase variation. To recover a spatially linear heating of the phantom, we fit the linear and constant phase distribution once on the region of interest (ROI) mask and once on only the signal from the reference tubes. Adding the first‐order fitted phase from the ROI and subtracting the B_0_ drift component that is extracted by a fit onto the reference tubes, will result in the corrected ΔT‐maps
**FIGURE S2** Comparison of applying background field removal (BFR) as a step for each time point before phase subtraction and applying BFR after phase subtraction, as suggested in Figure 2. A, Results for applying projection onto dipole fields (PDF) show that different solutions are found using PDF especially at the border of the phantom and the water bolus. At 2 time points, applying PDF before phase subtraction completely failed because the temperature‐induced phase change was misinterpreted as background phase. B, Results for applying Laplacian boundary value (LBV) show fewer difference between both approaches, indicating that it is a more robust approach for avoiding falsely subtracting temperature‐induced PRFS. However, different interpretation of smaller dipoles at the edge of the phantom could be noted, depending on the time point of the BFR application
**FIGURE S3** Treatment planning data for patient shown in Figure 9. The simulated normalized specific absorption rate (SAR) in % is overlaid on the CT data set. The target area is delineated with a red line. A, Axial view, B, Coronal view, C, Sagittal view

## References

[1] Kampinga HH . Cell biological effects of hyperthermia alone or combined with radiation or drugs: A short introduction to newcomers in the field. Int J Hyperthermia. 2006;22:191‐196. 16754338 10.1080/02656730500532028

[2] Gellermann J , Wlodarczyk W , Hildebrandt B , et al. Noninvasive magnetic resonance thermography of recurrent rectal carcinoma in a 1.5 Tesla hybrid system. Cancer Res. 2005;65:5872‐5880. 15994965 10.1158/0008-5472.CAN-04-3952

[3] McDannold N . Quantitative MRI‐based temperature mapping based on the proton resonant frequency shift: Review of validation studies. Int J Hyperthermia. 2005;21:533‐546. 16147438 10.1080/02656730500096073

[4] Ishihara Y , Calderon A , Watanabe H , et al. A precise and fast temperature mapping using water proton chemical shift. Magn Reson Med. 1995;34:814‐823. 8598808 10.1002/mrm.1910340606

[5] Dadakova T , Gellermann J , Voigt O , et al. Fast PRF‐based MR thermometry using double‐echo EPI: In vivo comparison in a clinical hyperthermia setting. MAGMA. 2015;28:305‐314. 25381180 10.1007/s10334-014-0467-y

[6] Gellermann J , Hildebrandt B , Issels R , et al. Noninvasive magnetic resonance thermography of soft tissue sarcomas during regional hyperthermia: Correlation with response and direct thermometry. Cancer. 2006;107:1373‐1382. 16902986 10.1002/cncr.22114

[7] Peters RD , Henkelman RM . Proton‐resonance frequency shift MR thermometry is affected by changes in the electrical conductivity of tissue. Magn Reson Med. 2000;43:62‐71. 10642732 10.1002/(sici)1522-2594(200001)43:1<62::aid-mrm8>3.0.co;2-1

[8] Winter L , Oberacker E , Paul K , et al. Magnetic resonance thermometry: Methodology, pitfalls and practical solutions. Int J Hyperthermia. 2016;32:63‐75. 26708630 10.3109/02656736.2015.1108462

[9] Schmitt A , Mougenot C , Chopra R . Spatiotemporal filtering of MR‐temperature artifacts arising from bowel motion during transurethral MR‐HIFU. Med Phys. 2014;41:113302. 25370670 10.1118/1.4897382 PMC4290727

[10] Streicher MN , Schafer A , Reimer E , et al. Effects of air susceptibility on proton resonance frequency MR thermometry. MAGMA. 2012;25:41‐47. 21479876 10.1007/s10334-011-0249-8

[11] Poorter JD . Noninvasive MRI thermometry with the proton resonance frequency method: Study of susceptibility effects. Magn Reson Med. 1995;34:359‐367. 7500875 10.1002/mrm.1910340313

[12] Baron P , Deckers R , de Greef M , et al. Correction of proton resonance frequency shift MR‐thermometry errors caused by heat‐induced magnetic susceptibility changes during high intensity focused ultrasound ablations in tissues containing fat. Magn Reson Med. 2014;72:1580‐1589. 24347129 10.1002/mrm.25063

[13] de Rochefort L , Liu T , Kressler B , et al. Quantitative susceptibility map reconstruction from MR phase data using bayesian regularization: Validation and application to brain imaging. Magn Reson Med. 2010;63:194‐206. 19953507 10.1002/mrm.22187

[14] Liu T , Khalidov I , de Rochefort L , et al. A novel background field removal method for MRI using projection onto dipole fields (PDF). NMR Biomed. 2011;24:1129‐1136. 21387445 10.1002/nbm.1670 PMC3628923

[15] He MZC , Tie C , Guo W , Chung YC , Liu X . Improving the temperature accuracy of referenceless MR thermometry in the presense of susceptibility artifact. In Proceedings of the 21st Annual Meeting of ISMRM, Salt Lake City, UT, 2013. Abstract 4292.

[16] Tan J , Mougenot C , Pichardo S , Drake JM , Waspe AC . Motion compensation using principal component analysis and projection onto dipole fields for abdominal magnetic resonance thermometry. Magn Reson Med. 2019;81:195‐207. 30058167 10.1002/mrm.27368

[17] Tan JWA , Mougenot C , Hynynen K , Drake JM , Pichardo S . Motion Compensation using Principal Component Analysis and Projection onto Dipole Fields for Abdominal Magnetic Resonance Thermometry during High‐Intensity Focused Ultrasound. In Proceedings of the 24th Annual Meeting of ISMRM, Singapore, 2016. Abstract 0818.

[18] Zhou D , Liu T , Spincemaille P , Wang Y . Background field removal by solving the Laplacian boundary value problem. NMR Biomed. 2014;27:312‐319. 24395595 10.1002/nbm.3064

[19] Schweser F , Robinson SD , de Rochefort L , Li W , Bredies K . An illustrated comparison of processing methods for phase MRI and QSM: removal of background field contributions from sources outside the region of interest. NMR Biomed. 2017;30:e3604. doi: 10.1002/nbm.3604. PMC5587182 27717080

[20] Ozbay PS , Deistung A , Feng X , Nanz D , Reichenbach JR , Schweser F . A comprehensive numerical analysis of background phase correction with V‐SHARP. NMR Biomed. 2017;30:e3550. doi:10.1002/nbm.3550. PMC5136354 27259117

[21] Sun H , Wilman AH . Background field removal using spherical mean value filtering and Tikhonov regularization. Magn Reson Med. 2014;71:1151‐1157. 23666788 10.1002/mrm.24765

[22] Li W , Avram AV , Wu B , Xiao X , Liu C . Integrated Laplacian‐based phase unwrapping and background phase removal for quantitative susceptibility mapping. NMR Biomed. 2014;27:219‐227. 24357120 10.1002/nbm.3056 PMC3947438

[23] Fortier V , Levesque IR . Phase processing for quantitative susceptibility mapping of regions with large susceptibility and lack of signal. Magn Reson Med. 2018;79:3103‐3113. 29130526 10.1002/mrm.26989

[24] El‐Sharkawy AM , Schär M , Bottomley PA , Atalar E . Monitoring and correcting spatio‐temporal variations of the MR scanner’s static magnetic field. Magn Reson Mater Phys, Biol Med. 2006;19:223‐236. 10.1007/s10334-006-0050-2 PMC1945237 17043837

[25] Shmatukha AV , Harvey PR , Bakker CJ . Correction of proton resonance frequency shift temperature maps for magnetic field disturbances using fat signal. J Magn Reson Imaging. 2007;25:579‐587. 17335067 10.1002/jmri.20835

[26] Wu M , Mulder HT , Zur Y , et al. A phase‐cycled temperature‐sensitive fast spin echo sequence with conductivity bias correction for monitoring of mild RF hyperthermia with PRFS. MAGMA. 2019;32:369‐380. 30515641 10.1007/s10334-018-0725-5

[27] Rieke V , Vigen KK , Sommer G , Daniel BL , Pauly JM , Butts K . Referenceless PRF shift thermometry. Magn Reson Med. 2004;51:1223‐1231. 15170843 10.1002/mrm.20090

[28] Grissom WA , Lustig M , Holbrook AB , Rieke V , Pauly JM , Butts‐Pauly K . Reweighted l1 referenceless PRF shift thermometry. Magn Reson Med. 2010;64:1068‐1077. 20564600 10.1002/mrm.22502 PMC3155729

[29] de Senneville BD , Roujol S , Moonen C , Ries M . Motion correction in MR thermometry of abdominal organs: A comparison of the referenceless vs. the multibaseline approach. Magn Reson Med. 2010;64:1373‐1381. 20677237 10.1002/mrm.22514

[30] Ferrer CJ , Bartels LW , van der Velden TA , et al. Field drift correction of proton resonance frequency shift temperature mapping with multichannel fast alternating nonselective free induction decay readouts. Magn Reson Med. 2020;83:962‐973. 31544289 10.1002/mrm.27985 PMC6899537

[31] Hernandez D , Kim KS , Michel E , Lee SY . Correction of B0 drift effects in magnetic resonance thermometry using magnetic field monitoring technique. Concepts Magn Reson Part B Magn Reson Eng. 2016;46B:81‐89.

[32] Li L , Leigh JS . Quantifying arbitrary magnetic susceptibility distributions with MR. Magn Reson Med. 2004;51:1077‐1082. 15122694 10.1002/mrm.20054

[33] Bowman RR . A probe for measuring temperature in radiofrequency‐heated material. IEEE T Microw Theory. 1976;24:43‐45.

[34] Bouwman JG , Bakker CJ . Alias subtraction more efficient than conventional zero‐padding in the Fourier‐based calculation of the susceptibility induced perturbation of the magnetic field in MR. Magn Reson Med. 2012;68:621‐630. 22711589 10.1002/mrm.24343

[35] de Zwart JA , Vimeux FC , Palussiere J , et al. On‐line correction and visualization of motion during MRI‐controlled hyperthermia. Magn Reson Med. 2001;45:128‐137. 11146494 10.1002/1522-2594(200101)45:1<128::aid-mrm1017>3.0.co;2-m

[36] Sprinkhuizen SM , Konings MK , van der Bom MJ , Viergever MA , Bakker CJ , Bartels LW . Temperature‐induced tissue susceptibility changes lead to significant temperature errors in PRFS‐based MR thermometry during thermal interventions. Magn Reson Med. 2010;64:1360‐1372. 20648685 10.1002/mrm.22531

[37] van Griethuysen JJM , Bus EM , Hauptmann M , et al. Gas‐induced susceptibility artifacts on diffusion‐weighted MRI of the rectum at 1.5T ‐ Effect of applying a micro‐enema to improve image quality. Eur J Radiol. 2018;99:131‐137. 29362144 10.1016/j.ejrad.2017.12.020

[38] Chu W , Staruch RM , Pichardo S , et al. Magnetic resonance‐guided high‐intensity focused ultrasound hyperthermia for recurrent rectal cancer: MR thermometry evaluation and preclinical validation. Int J Radiat Oncol Biol Phys. 2016;95:1259‐1267. 27209510 10.1016/j.ijrobp.2016.03.019

