# Supplementary material for: Correction of motion‐induced susceptibility artifacts and B0 drift during proton resonance frequency shift‐based MR thermometry in the pelvis with background field removal methods
Source: Magn Reson Med. 2020 May 5;84(5):2495–511. doi: 10.1002/mrm.28302 (PMC7402020; doi:10.1002/mrm.28302)
Supplement: Supplementary file 1 — FIGURE S1 Flow chart describing the pipeline for susceptibility artifact correction via background field removal algorithms using Double Echo Gradient Echo (DEGRE) data in case for the phantom heating data. For conductivity bias correction, the phase difference of long and short echo images is computed for the reference and current time point. The background field removal (BFR) is applied on the phase difference image of both phase differences, and removes susceptibility artifacts but also any spatially linear or constant phase variation. To recover a spatially linear heating of the phantom, we fit the linear and constant phase distribution once on the region of interest (ROI) mask and once on only the signal from the reference tubes. Adding the first‐order fitted phase from the ROI and subtracting the B0 drift component that is extracted by a fit onto the reference tubes, will result in the corrected ΔT‐maps FIGURE S2 Comparison of applying background field removal (BFR) as a step for each time point before phase subtraction and applying BFR after phase subtraction, as suggested in Figure 2. A, Results for applying projection onto dipole fields (PDF) show that different solutions are found using PDF especially at the border of the phantom and the water bolus. At 2 time points, applying PDF before phase subtraction completely failed because the temperature‐induced phase change was misinterpreted as background phase. B, Results for applying Laplacian boundary value (LBV) show fewer difference between both approaches, indicating that it is a more robust approach for avoiding falsely subtracting temperature‐induced PRFS. However, different interpretation of smaller dipoles at the edge of the phantom could be noted, depending on the time point of the BFR application FIGURE S3 Treatment planning data for patient shown in Figure 9. The simulated normalized specific absorption rate (SAR) in % is overlaid on the CT data set. The target area is delineated with a red line. A, [file MRM-84-2495-s001.pdf]

Correction of motion-induced susceptibility artifacts and B0 drift during proton resonance frequency shift-based MR thermometry in the pelvis with background field removal methods

Mingming Wu[1], Hendrik T. Mulder[2], Paul Baron[2], Eduardo Coello[1,3], Marion I. Menzel[3], Gerard C. van Rhoon[2] and Axel Haase[1]

[1]Munich School of Bioengineering, TUM Department of Physics, Technical University of Munich, Garching, Germany

[2]Erasmus MC Cancer Institute, Rotterdam, The Netherlands

[3]GE Global Research, Garching, Germany

Corresponding Author:

Mingming Wu, MSc
Munich School of Bioengineering
TUM Department of Physics
Technical University of Munich
Boltzmannstr. 11
D-85748 Garching b. München
Germany
E-mail: mingming.wu@tum.de
ORCiD 0000-0002-8593-7091

Keywords: MR thermometry, susceptibility, motion, background field removal, hyperthermia, B0 drift

Supporting Figure S1

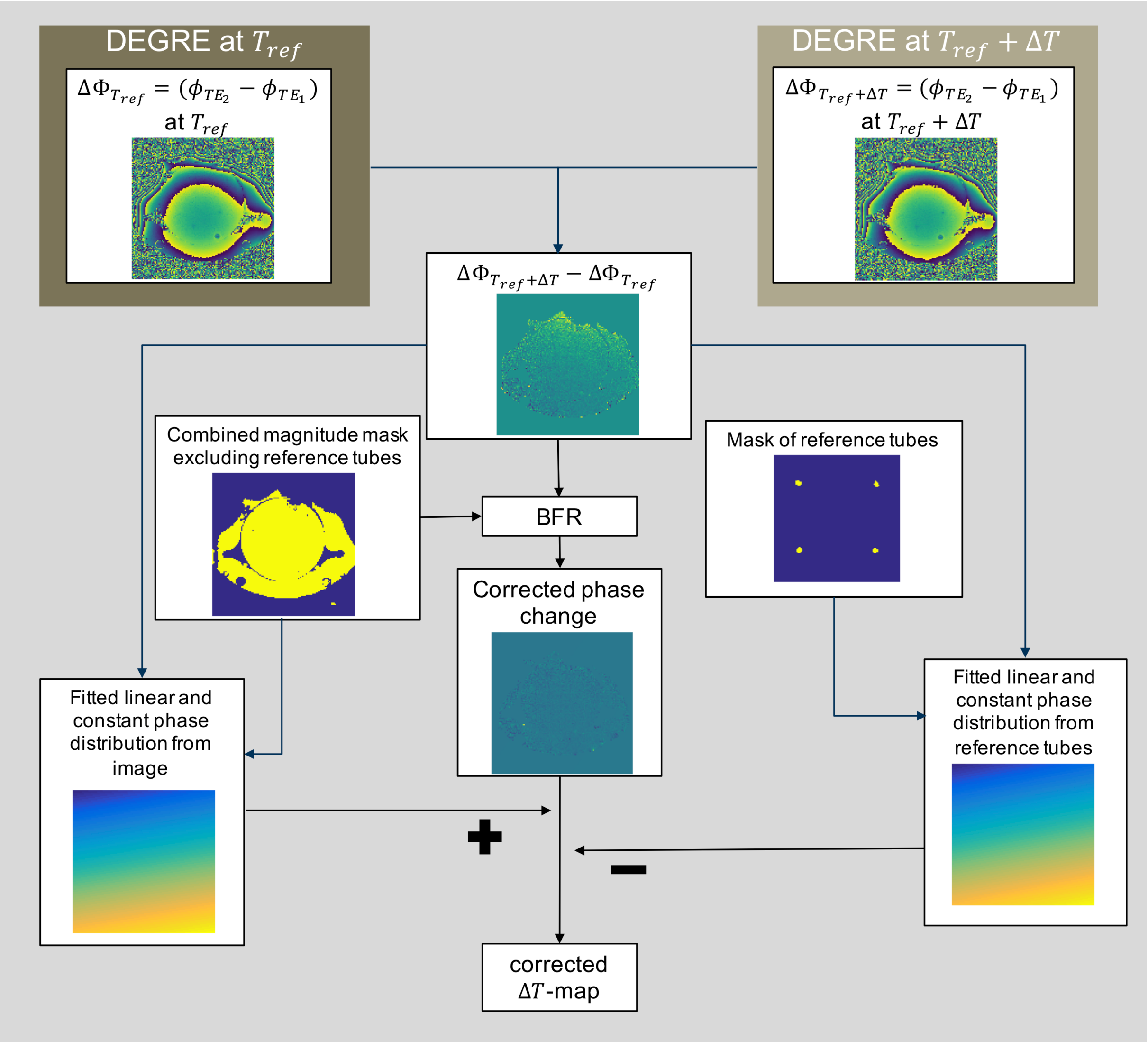

Flow chart describing the pipeline for susceptibility artefact correction via background field removal algorithms using DEGRE data in case for the phantom heating data. For conductivity bias correction, the phase difference of long and short echo images is computed for the reference and current time point. The BFR is applied on the phase difference image of both phase differences, and removes susceptibility artefacts, but also any spatially linear or constant phase variation. To recover a spatially linear heating of the phantom, we fit the linear and constant phase distribution once on the ROI mask and once on only the signal from the reference tubes. Adding the first order fitted phase from the ROI and subtracting the B0 drift component that is extracted by a fit onto the reference tubes, will result in the corrected ΔT-maps.

Supporting Figure S2

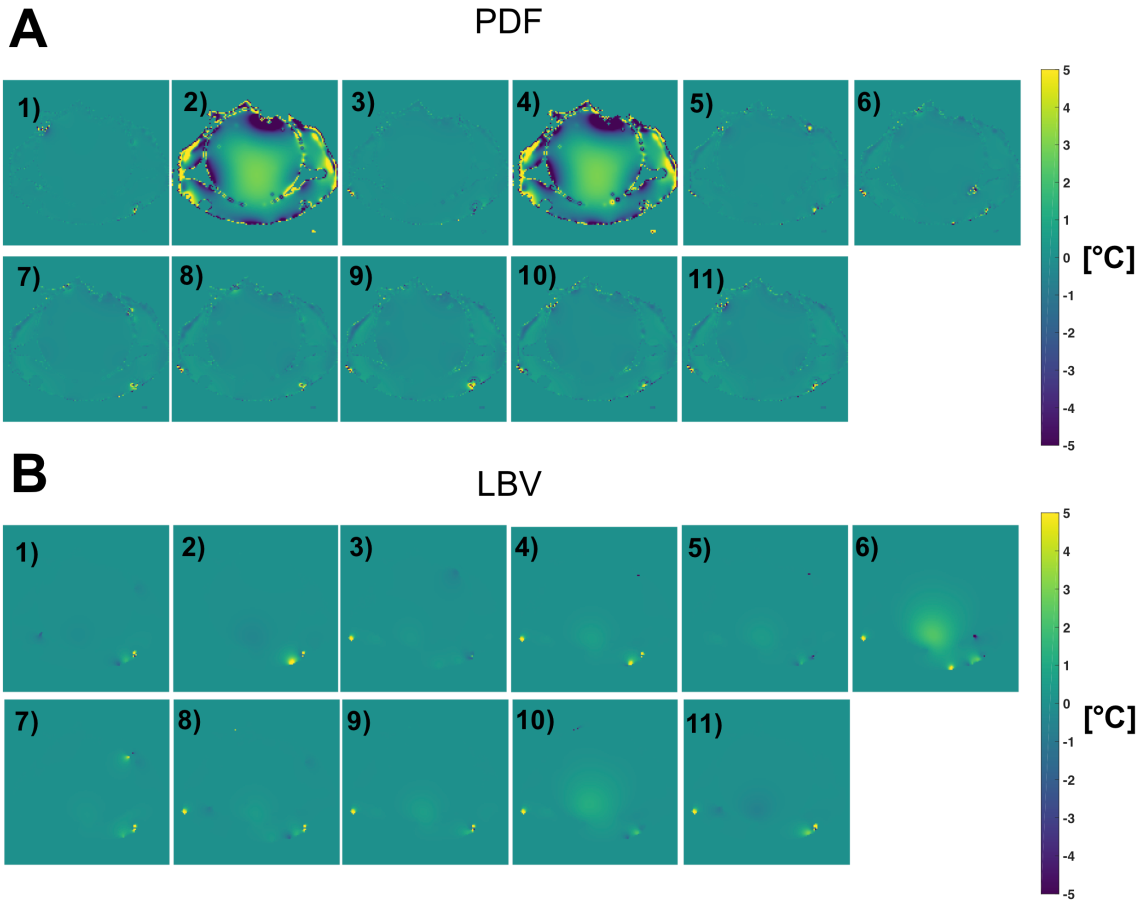

Comparison of applying BFR as a step for each time point before phase subtraction and applying BFR after phase subtraction, as suggested in Fig.2. A) Results for applying PDF show that different solutions are found using PDF especially at the border of the phantom and the water bolus. At two time points, applying PDF before phase subtraction completely failed because the temperature induced phase change was misinterpreted as background phase. B) Results for applying LBV show fewer difference between both approaches, indicating that it is a more robust approach for avoiding falsely subtracting temperature-induced PRFS. However, different interpretation of smaller dipoles at the edge of the phantom could be noted, depending on the time point of the BFR application.

Supporting Figure S3

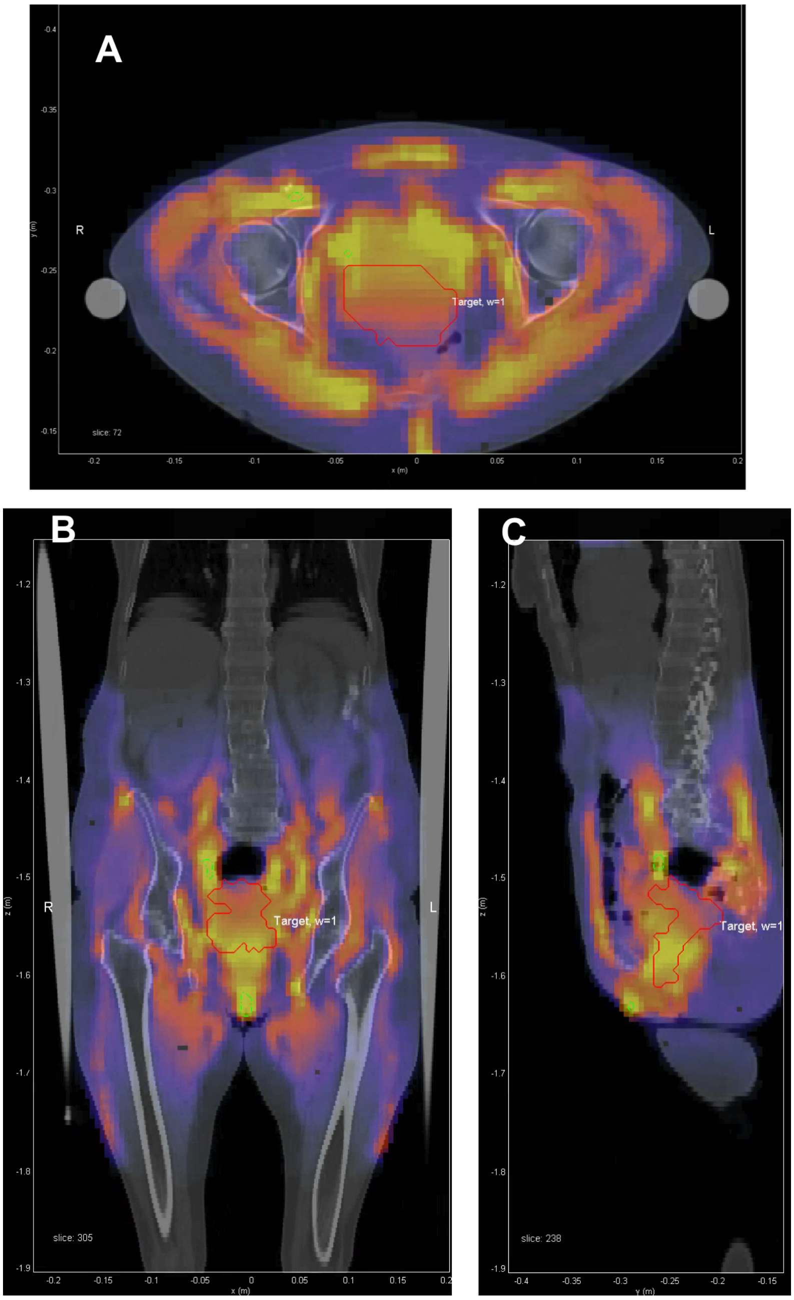

Treatment planning data for patient shown in Fig.9. The simulated normalized specific absorption rate (SAR) in % is overlaid on the CT dataset. The target area is delineated with a red line. A) Axial view, B) Coronal view, C) Sagittal view.
